# PF4 Silencing Promotes Trophoblast Cell Proliferation, Migration, Invasion and EMT by Regulating SOCS3/STAT3 Signaling Pathway

**DOI:** 10.2174/0118715303299470240723060939

**Published:** 2024-12-17

**Authors:** Hui Cao, Ranran Li, Li Ling, Guantai Ni

**Affiliations:** 1 Anhui Medical University, Hefei, Anhui, 230000, PR, China;; 2 Department of Obstetrics and Gynaecology, The First Affiliated Hospital of Wannan Medical College, Wuhu, Anhui, 241000, PR, China

**Keywords:** Recurrent miscarriage, PF4, SOCS3, STAT3, angiogenesis, spontaneous abortions (RSA)

## Abstract

**Background:**

Recurrent Miscarriage (RM) is a chronic and heterogeneous autoimmune disease. Platelet factor 4 (PF4) has been found to be involved in the pathogenesis of RM.

**Objective:**

We aimed to explore the role and mechanism of PF4 on trophoblasts in RM *in vitro*.

**Methods:**

In this study, the expression of PF4 and PF4 receptor CXC chemokine receptor 3 (CXCR3) in the placentas of patients with RM were analyzed by RT-qPCR and western blotting. Serum PF4 level was tested by ELISA. Following PF4 silencing and SOCS3 overexpression in HTR-8/SVneo cells, cell proliferation was detected by CCK-8, colony formation, and EDU assays. Wound healing and transwell assays separately evaluated cell migration and invasion. Immunofluorescence assay determined E-cadherin expression. Tube formation assay was used to measure the angiogenesis. Western blotting examined the expression of metastasis, epithelial-mesenchymal transition (EMT) and suppressor of cytokine signaling 3 (SOCS3)/signal transducer and activator of transcription 3 (STAT3) signaling-associated proteins.

**Results:**

The results revealed that PF4 displayed increased expression in placental villus tissues of RM patients. Serum PF4 level was also elevated in RM patients. PF4 silencing promoted the proliferation, migration, invasion, EMT, and angiogenesis of HTR-8/SVneo cells. Additionally, PF4 knockdown downregulated SOCS3 expression to activate STAT3 signaling. SOCS3 overexpression countervailed the effects of PF4 deficiency on HTR-8/SVneo cells.

**Conclusion:**

In summary, PF4 participated in the proliferation, migration, invasion, EMT and angiogenesis of trophoblast cells by modulating the SOCS3/STAT3 signaling pathway, indicating that targeting the PF4/SOCS3/STAT3 pathway could be a novel therapy for RM.

## 1 INTRODUCTION

Recurrent miscarriage (RM) refers to two or more consecutive spontaneous abortions (RSA), the occurrence of which may involve parental chromosome rearrangement and endocrine abnormalities, thrombosis disease, immune factors and, infectious diseases, *etc*. However, there is still a large proportion of patients with unexplained recurrent miscarriage (URSA), the pathogenesis of which remains unknown [1]. It has been reported that the development of trophoblast, embryonic differentiation, endometrial implantation and maternal-fetal immune regulation is a main factor for the success of early pregnancy, while the adhesion and invasion of trophoblasts outside the villus into the maternal uterine epithelium are the prerequisites for successful pregnancy [2]. During normal pregnancy, placental trophoblast cells have the ability to invade like tumor cells. However, in the pathological state of RM, promoting the proliferation, migration and invasion of trophoblast cells may contribute to the maintenance of normal embryo implantation, growth, and development [3].

PF4 (CXCL4) belongs to a small chemokine family. It has been reported that PF4 itself, its splicing variants, and its derivatives possess anti-tumor, anti-migration, and anti-angiogenesis activities [4]. A recent study has reported that PF4 level is significantly elevated in the plasma of patients with RM [5]. Besides, STAT3 plays an important role in recurrent abortion, and inhibition of STAT3 expression can inhibit the proliferation and migration of trophoblast cells [6]. Additionally, PF4 has been found to increase SOCS3 in tumor cells, thereby inhibiting the level of STAT3 and activating inflammation [7]. As an inhibitor of the STAT3 pathway, SOCS3 plays a crucial role in inhibiting STAT3 expression in tumor angiogenesis, lung injury, and other aspects [8, 9]. Therefore, this study aims to explore whether PF4 regulates STAT3 through SOCS3 and affects trophoblast cell proliferation, migration, invasion and tubule formation so as to reveal the pathogenesis of URSA.

## 2 MATERIALS AND METHODS

### 2.1 Clinical Sample

Tissues samples were collected from 7 patients with a history of more than two consecutive pregnancy losses of unknown etiology (RSA group) and of 7 healthy patients with a legal termination of their pregnancy (control group) in The First Affiliated Hospital of Wannan Medical College. All placentas without any prior hormonal treatment were collected within the first 24 hours after diagnosis. The protocols of this study were conducted in accordance with the Ethical Review Committees of The First Affiliated Hospital of Wannan Medical College (approval number: ChiCTR2000032249). The study has obtained written consent from patients according to the policies of the committee. Experiments were in accordance with the ethical standards of the committee responsible for human experimentation (institutional national), and with the Helsinki Declaration of 1975, as revised in 2008.

### 2.2 Cell Culture and Plasmid Transfection

HTR-8/SVneo cells were purchased from ATCC and cultured in RPMI-1640 medium (Macgene, Beijing, China) containing 5% FBS (Macgene, Beijing, China) with 5% CO_2_ at 37°C. PF4 silencing (shRNA-PF4-1/2) or SOCS3 overexpression plasmids (Ov-SOCS3) and their negative control shRNA-NC and Ov-NC were constructed by GenePharma (Shanghai, China). The aforementioned plasmids were transfected into HTR-8/SVneo cells using Lipofectamine 3000 (Invitrogen). 48 h after transfection, cells were collected for further experiments.

### 2.3 Cell Counting Kit 8 (CCK8) Assay

HTR-8/SVneo cells at the logarithmic growth stage were digested, counted and inoculated into 96-well plates (1x10^4^ cells/ well, 100 µL/well). 10 μL CCK8 (Beijing Solarbio Science & Technology, Beijing, China) was added to each well for 4 h incubation. The absorbance was measured at 450 nm with a microplate reader (DNM-9602G, PERLONG Co., Ltd., Beijing, China).

### 2.4 Colony Formation Assay

HTR-8/SVneo cells were seeded into a 6-well plate containing 10 mL of culture solution (1x10^3 ^cells per well). The plate was gently rotated. The cells were evenly placed in a culture incubator with 5% CO_2_ at 37°C for 14 days. The cells were washed twice with PBS and fixed with 4% paraformaldehyde for 15 min. Then, crystal violet solution was added for dyeing for 10~30 min. Finally, the number of colonies was counted manually.

### 2.5 EDU Staining

HTR-8/SVneo cells were seeded into a 96-well plate (1x10^5^ cells/well). 20 µmol/l of diluted EDU (Nanjing KeyGen Biotech, Co., Ltd.) was added into each well for incubation for 2 h. Next, cells were immobilized by 4% paraformaldehyde for 15 min and permeated with 0.5% Triton X-100 for 10 min at room temperature, followed by incubation with prepared Click-iT reaction cocktail at room temperature for 30 min in the dark. Nuclear staining was performed with DAPI for 30 min. The images were observed under a fluorescence microscope (Olympus Corporation).

### 2.6 Wound Healing Assay

HTR-8/SVneo cells in the logarithmic growth period were digested and counted before inoculation in 6-well plates (4x10^5^ cells/well). When the cell confluence reached 90%, cells were scratched vertically with a 200 μL pipette tip and rinsed with PBS. After 24 h, the migration distance of cells in each group was photographed and observed by an inverted microscope (Olympus Corporation).

### 2.7 Transwell Assay

Transwell chambers coated with Matrigel (BD Biosciences) were used to detect the invasion of HTR-8/SVneo cells. Cells digested at the logarithmic growth stage were counted and seeded into Transwell chamber (2x10^4^ cells/well) with serum-free medium. 750 μL 10% FBS was added to the medium in the lower chamber, and the cells were cultured in the medium for 24 h. Then, cells were fixed with 4% paraformaldehyde for 15 min and stained with 0.1% crystal violet for 15 min. The images were photographed using an inverted light microscope (Olympus Corporation).

### 2.8 Enzyme-linked Immunosorbent Assay (ELISA)

The serum was harvested from the blood samples by centrifugation at 1,500 x g at 4°C for 15 min. The serum PF4 level was examined with a human PF4 ELISA kit (ab278096; Abcam). The optical density values were detected using a microplate reader (DNM-9602G, PERLONG Co., Ltd., Beijing, China).

### 2.9 Western Blotting

After cells were lysed in RIPA buffer, the total protein concentration was detected using the BCA method. The protein lysates (25 µg protein per lane) were separated by a 10% SDS-PAGE gel for electrophoresis. The separated proteins were then transferred to the PVDF films. After 2 h blocking within 5% BSA, these membranes were incubated with primaries antibodies overnight at 4°C. The horseradish peroxidase-labeled secondary antibody (cat. no. 7074P2; Cell Signaling Technology, Boston, MA, USA) was incubated successively with the membranes. Membranes were visualized using a chemiluminescence reagent (Bio-rad, USA). ImageJ software 1.4.3 (National Institutes of Health) was used to analyze the gray values of protein bands. GAPDH was used as a loading control. Anti-PF4 (cat. no. 21157-1-AP), anti-CXCR3 (cat. no. 26756-1-AP), anti- matrix metalloproteinase-2 (MMP2) (cat. no. 10373-2-AP), anti-MMP9 (cat. no. 27306-1-AP), anti-E-cadherin (cat. no. 20874-1-AP), anti-N-cadherin (cat. no. 22018-1-AP), anti-Vimentin (cat. no. 10366-1-AP) and anti-SOCS3 (cat. no. 14025-1-AP) antibodies were provided by Proteintech (Chicago, IL, USA). Anti-phospho (p)-STAT3 (tyr705; cat. no. 9145T), anti-STAT3 (cat. no. 4904T) and anti-GAPDH (cat. no. 4904T) antibodies were obtained from Cell Signaling Technology (Boston, MA, USA).

### 2.10 Immunofluorescence Assay

HTR-8/SVneo cells were seeded in a 24-well plate. After 48 h incubation, cells were washed with PBS 3 times and fixed with 4% paraformaldehyde for 10 min. Cells were incubated with 0.5% Triton X-100, washed with PBS for 2 min at 4°C and blocked with 5% BSA at room temperature for 1 h. After that, HTR-8/SVneo cells were incubated with an anti-E-cadherin antibody (cat. no. 20874-1-AP; Proteintech, Chicago, IL, USA) at 4°C overnight. The Alexa Fluor-488 conjugated secondary antibody (cat. no. 4412S; Cell Signaling Technology, Boston, MA, USA) was employed to incubate the slides for 1 h. The nuclei were stained with DAPI at room temperature for 5 min. The immunofluorescence was visible under a fluorescence microscope (Olympus Corporation).

### 2.11 Tube Formation Assay

A total of 1.5x10^4^ HUVECs were seeded into a 96-well plate pre-coated with 50 µl/well Matrigel, which was then incubated with the supernatants from transfected HTR-8/SVneo cells. Tube formation was observed under an inverted light microscope (Olympus Corporation).

### 2.12 RT-qPCR Assay

Total RNA was extracted using the TRIzol^®^ reagent (Invitrogen, USA). According to the instructions of the RevertAid First Strand cDNA Synthesis Kit (Guangzhou Magen Biotechnology Co., Ltd), the cDNA was synthesized from total RNA. Reactions were run on an ABI 7500 Real-Time PCR System with the Maxima SYBR Green qPCR Master Mix (Guangzhou Magen Biotechnology Co., Ltd) following the product instructions. The following thermocycling conditions were used: Initial denaturation at 95°C for 10 min, followed by 40 cycles of denaturation at 95°C for 15 sec and annealing at 60°C for 1 min, and a final extension of 10 min at 72°C. Three multiple wells were set for each group. The experiment was repeated three times, and the expression level of the target gene was calculated using the 2^-∆∆Ct^ method. GAPDH was chosen to be the endogenous control for the calculation.

### 2.13 Statistical Analysis

All experiments were repeated three times independently. GraphPad Prism 8.0 was used for statistical processing. Data were expressed as the mean ± standard deviation. The Student’s T-test calculated the differences between the two groups. One-way ANOVA followed by Tukey’s hoc test was used to compare the groups. *P* less than 0.05 was supposed to be statistically significant.

## 3 RESULTS

### 3.1 PF4 Expression was Increased in the Placental Villus Tissues and Serum of RM Patients

**Fig. (1):**
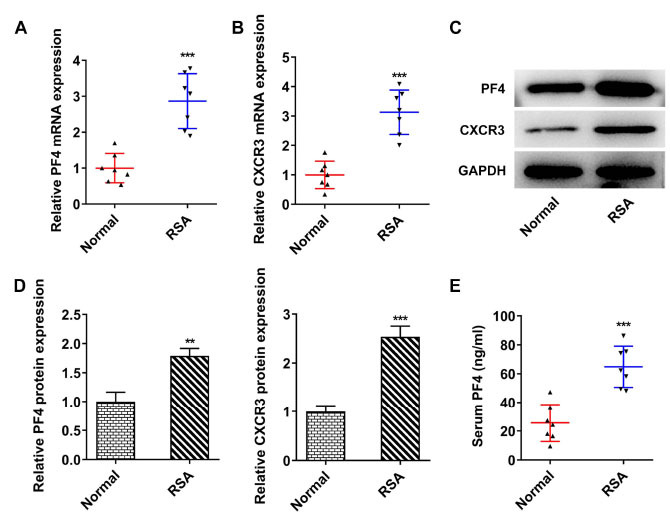
PF4 expression was increased in the placental villus tissues and serum of RM patients. (**A**) PF4 and (**B**) CXCR3 mRNA expression in placental villous tissues of RM patients (n = 7) and normal women (n = 7) was detected using RT-qPCR. (**C**-**D**) PF4 and CXCR3 expression in placental villous tissues of RM patients (n = 7) and normal women (n = 7) was detected using western blotting. (**E**) The serum levels of PF4 were examined by ELISA. **P <0.01 and ***P <0.001 *vs*. Normal group.

To determine the expression of PF4 and PF4 receptor CXCR3 in the placental villus tissues of patients with RM, RT-qPCR and western blotting were applied. The results indicated that the mRNA and protein levels of both PF4 and CXCR3 were elevated in the placentas of patients with RM compared with the placental villus tissues of healthy controls (Figs. **1A**-**D**). Besides, it was also discovered that PF4 levels in the serum of RM patients were higher than those in the normal group (Fig. **1E**).

### 3.2 PF4 Silencing Promoted the Proliferation of HTR-8/SVneo Cells

**Fig. (2):**
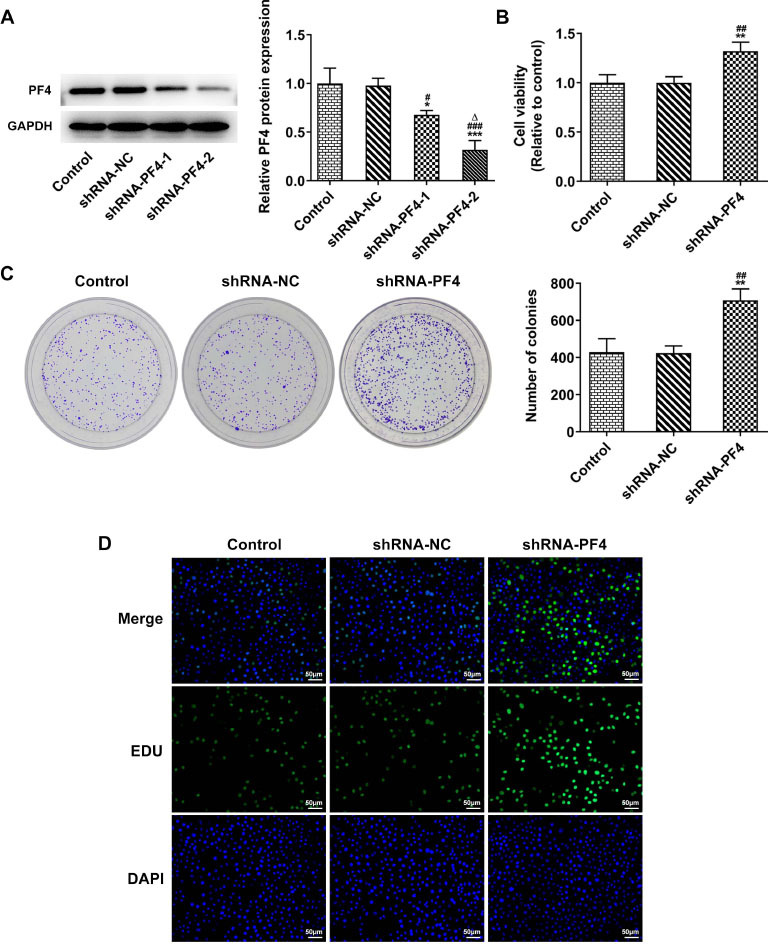
PF4 silencing promoted the proliferation of HTR-8/SVneo cells. (**A**) The transfection efficacy of PF4 interference plasmids in HTR-8/SVneo cells were measured using western blotting. (**B**) CCK-8 assay was used to detect cell viability. (**C**) Colony formation assay was used to measure cell colony-forming ability. (**D**) EDU staining was used to evaluate cell proliferation. *P <0.05 and **P <0.01 *vs*. Control group. ^#^*P* <0.05, ^##^*P* <0.01 and ^###^*P* <0.001 *vs*. shRNA-NC group. ^∆^*P* <0.05 *vs*. shRNA-PF4-1 group.

To explore the impacts of PF4 on trophoblast cells, PF4 was silenced by transfection with shRNA-PF4-1/2. Significantly downregulated PF4 expression was observed in the shRNA-PF4-1 and shRNA-PF4-2 groups compared with the shRNA-NC group (Fig. **2A**). Further, the interference efficacy of shRNA-PF4-2 was more significant than shRNA-PF4-1, hence being selected for the follow-up experiments. A CCK-8 assay was used to assess the viability of HTR-8/SVneo cells after transfection. It was noted that the absence of PF4 prominently potentiated the viability of HTR-8/SVneo cells as comparison to the shRNA-NC group (Fig. **2B**). Besides, the effect of PF4 insufficiency on the proliferation of HTR-8/SVneo cells was detected by using colony formation assay. As shown in Fig. (**2C**), PF4 down-regulation increased the colony-forming ability of HTR-8/SVneo cells. It was observed that PF4 silencing notably enhanced the EDU fluorescence intensity when compared with the shRNA-NC group, further suggesting the promoting effect of PF4 knockdown on the proliferation of HTR-8/SVneo cells (Fig. **2D**).

### 3.3 PF4 Silencing Promoted the Invasion, Migration, EMT and Tube Formation in HTR-8/SVneo Cells

**Fig. (3):**
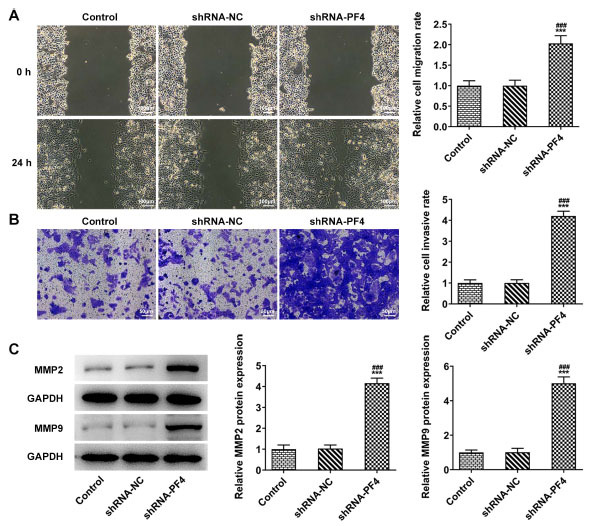
PF4 silencing promoted the invasion and migration in HTR-8/SVneo cells. (**A**) Wound healing assay was used to assess cell migration. (**B**) Transwell assay was used to detect cell invasion. (**C**) Western blotting was used to examine MMP2 and MMP9 expression. ***P <0.001 *vs*. Control group. ^###^*P* <0.001 *vs*. shRNA-NC group.

**Fig. (4):**
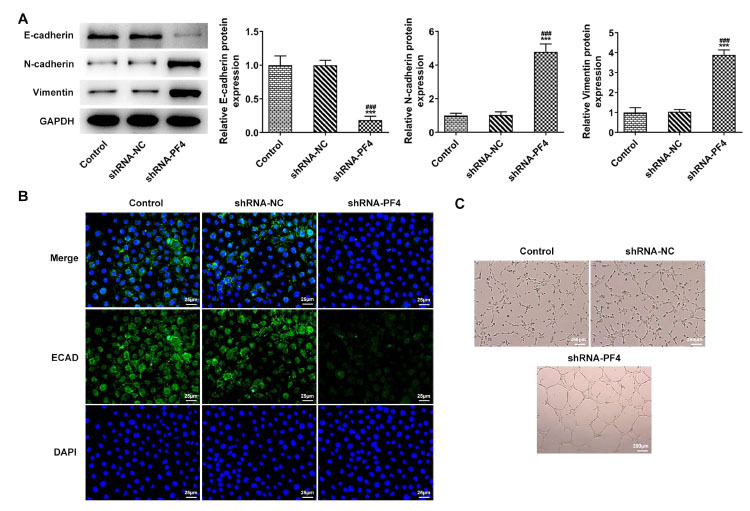
PF4 silencing promoted the EMT and tube formation in HTR-8/SVneo cells. (**A**) Western blotting was used to detect E-cadherin, N-cadherin, Vimentin expression. (**B**) Representative images of immunofluorescence assay for E-cadherin. (**C**) Tube formation assay was used to detect cell angiogenesis. ***P <0.001 *vs*. Control group. ^###^*P* <0.001 *vs*. shRNA-NC group.

At the same time, the results from wound healing and transwell assays presented that after PF4 was silenced, the migratory and invasive capacities of HTR-8/SVneo cells were both exacerbated (Figs. **3A** and **B**). These findings were also evidenced by the upregulated expression of metastasis-related MMP2 and MMP9 in the shRNA-PF4 group by contrast with that in the shRNA-NC group (Fig. **3C**). In addition, the expression levels of EMT-related markers were analyzed and it was noticed that E-cadherin expression was decreased while N-cadherin and Vimentin expression was increased in the shRNA-PF4 group compared with the shRNA-NC group (Figs. **4A** and **B**). Subsequently, tube formation was performed, and it was observed that PF4 deficiency resulted in increased tube formation in the shRNA-PF4 group compared with the shRNA-NC group (Fig. **4C**).

### 3.4 PF4 Silencing Inhibited SOCS3 and Thus Activated STAT3 Expression in HTR-8/SVneo Cells

**Fig. (5):**
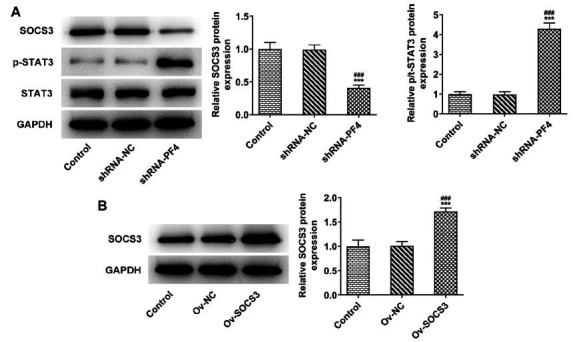
PF4 silencing inhibited SOCS3 and thus activated STAT3 expression. (**A**) Western blotting was used to detect SOCS3 and p/t-STAT3 expression. (**B**) Western blotting was used to examine the transfection efficacy of SOCS3 overexpression plasmids. ***P <0.001 *vs*. Control group. ^###^*P* <0.001 *vs*. shRNA-NC group.

To explore the mechanism underlying the regulatory effects of PF4 in HTR-8/SVneo cells, the expression of SOCS3/STAT3 signaling-associated proteins was tested. As depicted in Fig. (**5A**), SOCS3 expression level was decreased, whereas p/t-STAT3 expression level was increased after PF4 deficiency compared with the shRNA-NC group. To substantiate the PF4/SOCS3/STAT3 signaling in trophoblast cells, SOCS3 was overexpressed by transfection with Ov-SOCS3 plasmid, and significantly upregulated SOCS3 expression was found in the Ov-SOCS3 group (Fig. **5B**).

### 3.5 SOCS3 Overexpression Reversed the Effects of PF4 Silencing on the Proliferation, Invasion, Migration, EMT and Tube Formation in HTR-8/SVneo Cells

**Fig. (6):**
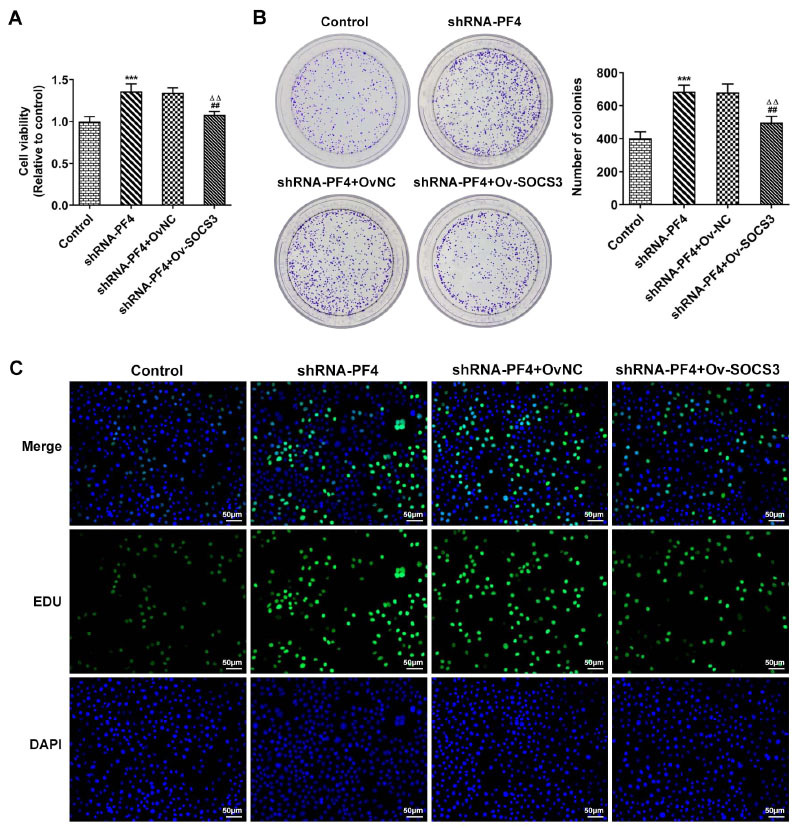
SOCS3 overexpression reversed the effects of PF4 silencing on the proliferation in HTR-8/SVneo cells. (**A**) CCK-8 assay was used to detect cell viability. (**B**) Colony formation assay was used to detect cell colony-forming ability. (**C**) EDU staining was used to evaluate cell proliferation. ***P <0.001 *vs*. Control group. ^##^*P* <0.01 *vs*. shRNA-PF4 group. ^∆∆^*P* <0.01 *vs*. shRNA-PF4+ Ov-SOCS3group.

**Fig. (7):**
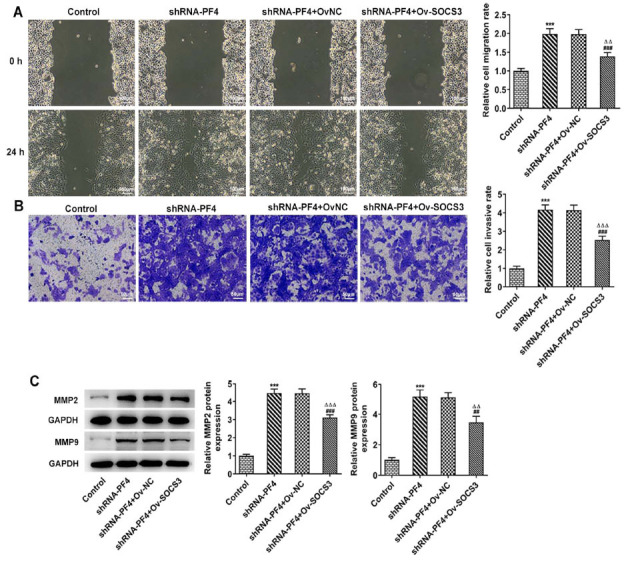
SOCS3 overexpression reversed the effects of PF4 silencing on the invasion and migration in HTR-8/SVneo cells. (**A**) Wound healing assay was used to detect cell migration. (**B**) Transwell assay was used to detect cell invasion. (**C**) Western blotting was used to detect MMP2 and MMP9 expression. ***P <0.001 *vs*. Control group. ^##^*P* <0.01 and ^###^*P* <0.001 *vs*. shRNA-PF4 group. ^∆∆^*P* <0.01, ^∆∆∆^*P* <0.001 *vs*. shRNA-PF4+ Ov-SOCS3group.

**Fig. (8):**
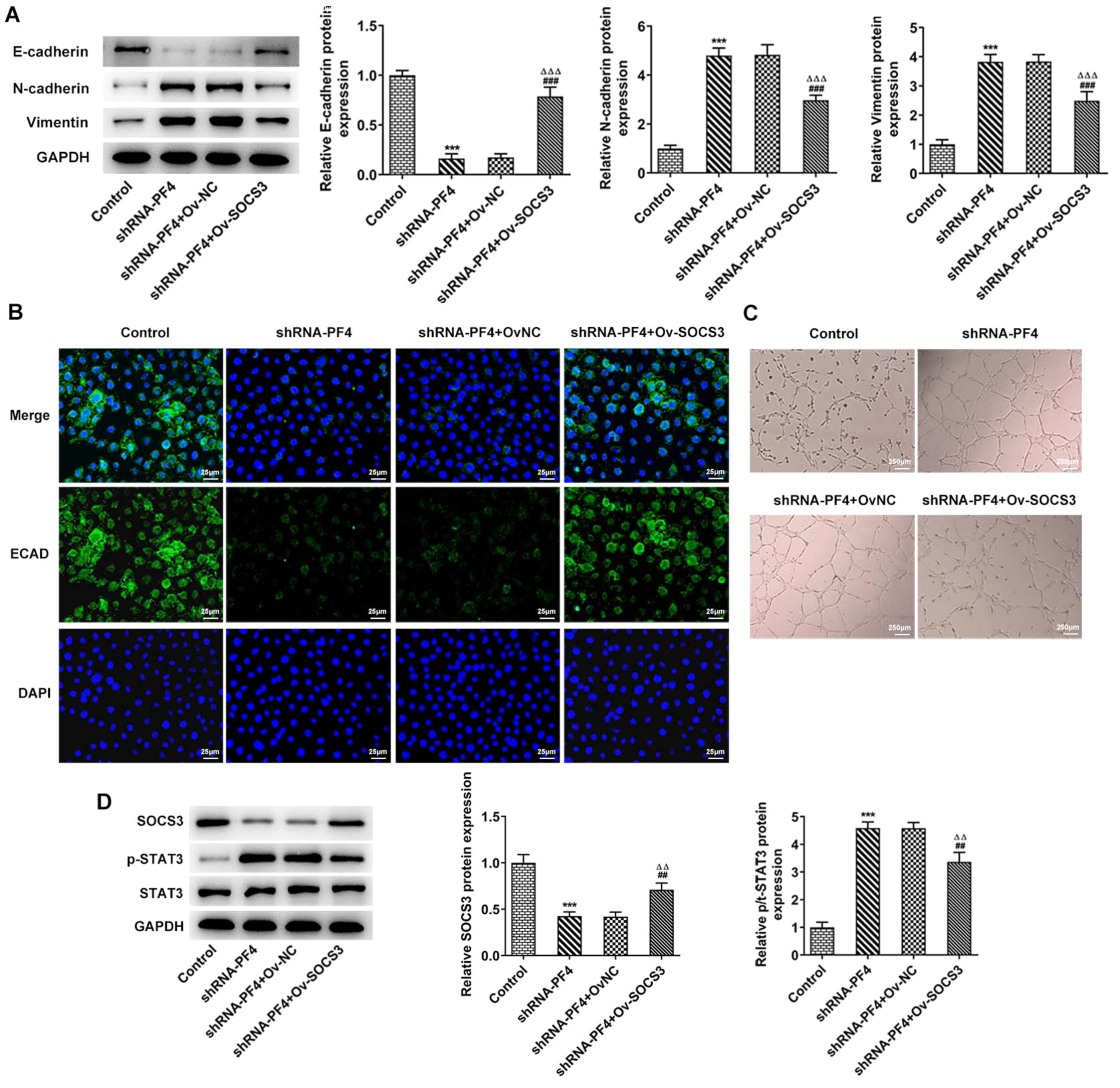
SOCS3 overexpression reversed the effects of PF4 silencing on the EMT and tube formation in HTR-8/SVneo cells. (**A**) Western blotting was used to detect E-cadherin, N-cadherin, Vimentin expression. (**B**) Representative images of immunofluorescence assay for E-cadherin. (**C**) Tube formation assay was used to detect cell angiogenesis. (**D**) Western blotting was used to measure SOCS3 and p/t-STAT3 expression. ***P <0.001 *vs*. Control group. ^##^*P* <0.01 and ^###^*P* <0.001 *vs*. shRNA-PF4 group. ^∆∆^*P* <0.01, ^∆∆∆^*P* <0.001 *vs*. shRNA-PF4+ Ov-SOCS3group.

As expected, CCK-8 assay delineated that the improved viability of HTR-8/SVneo cells transfected with PF4 interference plasmids was diminished when SOCS3 was up-regulated (Fig. **6A**). Similarly, the results from colony formation assay and EDU staining proved that overexpression of SOCS3 reversed the enhanced proliferation ability of HTR-8/SVneo cells caused by PF4 knockdown (Figs. **6B** and **C**). Moreover, SOCS3 overexpression also restored the impacts of PF4 silencing on the migration and invasion of HTR-8/SVneo cells (Figs. **7A** and **B**). Concurrently, western blotting and immunofluorescence staining showed that relative to the shRNA-PF4+Ov-NC group, E-cadherin expression was increased while MMP2, MMP9, N-cadherin and Vimentin expression was decreased in the shRNA-PF4+Ov-SOCS3 group (Figs. **7C**, **8A** and **B**). Similarly, the accelerated tube formation ability of HTR-8/SVneo cells on account of PF4 knockdown was blocked by SOCS3 overexpression (Fig. **8C**). Moreover, PF4 knockdown increased p/t-STAT3 expression level in HTR-8/SVneo cells, which was decreased after the further Ov-SOCS3 transfection (Fig. **8D**).

## 4 DISCUSSION

The placental villous tissue is composed of trophoblasts that are indispensable for embryonic and fetal growth as well as the overall function of the placenta [10]. Accordingly, trophoblast injury can damage the structure and function of placental villous tissues, potentially contributing to RM. The invasion of placental trophoblast cells into the maternal decidua and myometrium is critical for placental embedment and fetal development. A large number of studies have shown that abnormal proliferation, differentiation and apoptosis of placental trophoblast cells can result in decreased invasion ability of trophoblast cells, thereby causing repeated spontaneous abortion, preeclampsia, fetal intrauterine growth restriction and other pregnancy-associated diseases [11-13]. Hence, facilitating trophoblast proliferation, migration and invasion may be beneficial for RM treatment.

PF4 is a naturally occurring 70-amino acid protein that has been proposed to protect against human malignancies by inducing cell apoptosis [14]. More importantly, Plasma PF4 levels were significantly increased in RM patients [5]. Consistently, in this study, it was discovered that PF4 expression was increased in the placental villus tissues and serum of RM patients. As reported, PF4 inhibited the proliferation, migration, and tubulogenesis of monkey retinal vascular endothelial cells treated with vascular endothelial growth factor and tumor necrosis factor-alpha [15]. Nguyen *et al*. demonstrated that PF4 addition suppresses the CXCL12-induced migration of breast cancer cells [16]. PF4 has been proposed as a target for cancer therapy and a potential clinical anti-tumor agent [17]. In this study, the subsequent loss-of-function experiments expounded that PF4 knockdown notably increased the viability, proliferation, migration and invasion of HTR-8/SVneo cells. Specifically, MMP2 and MMP9 are well known to be capable of efficiently remodeling the extracellular matrix to facilitate the invasion capacity of trophoblasts [18]. The present study revealed that the expression levels of metastasis-related MMP2 and MMP9 were upregulated following PF4 deficiency. Many studies have suggested that EMT is capable of facilitating the invasion and migration of extravillous trophoblast cells [19, 20]. In addition, E-cadherin expression was decreased, while N-cadherin and Vimentin expression were increased when PF4 was knocked down in HTR-8/SVneo cells. Abnormal angiogenesis has been recognized to be associated with RM [21, 22]. As reported, PF4 has been deemed as an inhibitor of angiogenesis [23]. Here, the experimental data also illuminated that PF4 deficiency strengthened the tube-forming ability of HTR-8/SVneo cells.

STAT3 is a prototypical member of the STAT family that has been implicated in the process of RM [24]. Fang *et al*. have also supported that STAT3 expression is downregulated in abortion tissues in patients with early miscarriage and inhibition of STAT3 obstructs trophoblast cell proliferation [6]. STAT3 activation by phosphorylation is frequently observed during the malignant transformation into cancer [25]. Through inhibiting the activation of the STAT3 pathway, NINJ1 contributes to the development of recurrent spontaneous abortion and triggers trophoblast cell dysfunction [26]. Of note, SOCS3 has been identified as a feedback inhibitor of JAK/STAT3 signaling, which is involved in the signal transduction of many cytokines during many cellular processes [27, 28]. Intriguingly, it has been reported that SOCS3 elevation stimulates the proliferation of trophoblasts [29]. A growing body of evidence has confirmed that PF4 regulates the expression of SOCS3 in liver transplantation and ischemia-reperfusion injury in melanoma [7, 30]. Furthermore, Liang *et al*. have proposed that PF4 induces SOCS3 expression while negatively modulating STAT3 expression in multiple myeloma [31]. During this research, SOCS3 expression was downregulated, whereas p/t-STAT3 expression was upregulated when PF4 was knocked down, suggesting that PF4 silencing might suppress SOCS3 expression to activate STAT3 signaling in trophoblast cells. In particular, cellular behaviors, such as invasion, proliferation, migration, and EMT, have been found to be related to SOCS3/STAT3 pathway [32, 33]. At the same time, the present experimental data clarified that following overexpression of SOCS3, the potentiated trophoblast viability, proliferation, migration, invasion, EMT and angiogenesis on account of down-regulation of PF4 were attenuated. These results demonstrated that the dysregulation of the PF4/SOCS3/STAT3 signaling pathway might play a vital role in the pathogenesis of RM by affecting trophoblast development.

## CONCLUSION

In the present study, we found that the expression level of PF4 was significantly upregulated in the villus tissues of patients with RM, and insufficiency of PF4 contributed to the proliferation, migration, invasion, EMT, and tube formation of trophoblasts by inhibiting SOCS3 to activate STAT3 signaling. This study might suggest a potential role of PF4/SOCS3/STAT3 signaling in the progression of RM. These novel findings shed light on the role of PF4 in the regulation of trophoblast behaviors in the prevention and treatment of RM.

## AUTHORS’ CONTRIBUTIONS

The authors confirm their contribution to the paper as follows: study conception and design: H.C., G.N.; data collection: R.L.; data analysis and interpretation: L.L. All authors reviewed the results and approved the final version of the manuscript.

## Data Availability

The datasets used and/or analyzed during the current study are available from the corresponding author upon reasonable request.

## LIST OF ABBREVIATIONS

CCK8: Cell Counting Kit 8
CXCR3: CXC Chemokine Receptor 3
ELISA: Enzyme-linked Immunosorbent Assay
PF4: Platelet Factor 4
RM: Recurrent Miscarriage
